# Estimating c-section coverage: Assessing method performance and characterizing variations in coverage

**DOI:** 10.7189/jogh.21.08002

**Published:** 2022-04-09

**Authors:** Emily D Carter, P Neff Walker

**Affiliations:** Johns Hopkins School of Public Health, Baltimore, Maryland, USA

## Abstract

**Background:**

Cesarean section (c-section) is an essential tool for preventing, stillbirths, maternal, and newborn death. However, data on coverage of medically necessary c-section is limited in low- and middle-income settings.

**Methods:**

We estimated national c-section coverage using household survey data from 98 low- and middle-income countries. To disaggregate elective and medically necessary c-sections, we estimated the proportion of women in each survey wealth quintile who gave birth via c-section assuming a denominator that 12.5% of births necessitate a c-section delivery. We capped stratum coverage at 100%. We estimated national c-section coverage weighting for the proportion of births occurring in each wealth quintile. We examined 1) variation in estimated c-section by wealth quintile, national income classification, and stage in the obstetric transition, 2) how varying definitions impact the classification of countries' access to c-section, and 3) correlation between c-section and related mortality outcomes.

**Results:**

Both increasing national and household wealth are associated with increasing levels of c-section coverage and c-section rate. C-section coverage was highly inequitable by wealth within a country. Differentials in coverage were most pronounced in countries with c-section rates below 10%; however, some countries showed significant gaps in c-section coverage in poor subpopulations despite high c-section rates nationally. The choice of indicator and threshold altered whether a country was classified as having adequate access to c-section services. C-section coverage estimates showed a stronger relationship with closely related health outcomes than national c-section rates.

**Conclusions:**

Generating estimates of c-section coverage is crucial for gauging gaps in c-section access. Our approach for calculating c-section coverage using stratification by wealth to adjust for potential elective c-sections is supported by the strong correlations between household wealth and subnational c-section rate, and the association between our coverage estimates and health outcomes at a national level. Looking at national c-section rates alone may paint an inaccurate picture of c-section access and mask subnational inequities in coverage. The need to accurately measure access to c-section will continue to increase as growth in LMICs drives inequities in coverage and introduces dual concerns related to c-section overuse in some populations while others lack access to care.

Cesarean section (c-section) is a life-saving intervention and one of the most common surgical procedures performed globally. C-sections can prevent maternal deaths, intrapartum stillbirths, and early neonatal deaths due to hemorrhage, fetal stress or malpresentation, and hypertension [1]. C-sections are surgical procedures and require a woman in need to access an appropriately trained and supplied facility promptly. Ensuring equitable access to this essential intervention is key to continued reductions in maternal and neonatal mortality.

Current guidance from the World Health Organization (WHO) considers 10%-15% to be the ideal c-section rate [2]. Estimates of the c-section rate in most low- and middle-income countries (LMICs) are derived from nationally representative household surveys in which women are asked to report on the delivery method for their most recent birth. However, these data do not capture information to ascertain whether a c-section was medically necessary. C-section rates have doubled globally since 2000 and in many LMICs exceed the 10%-15% rate, introducing concern about overuse [1]. Elective c-sections and other non-medically indicated c-sections carry an increased risk of infection, may necessitate c-section for future births, and reduce beneficial breastfeeding practices compared to vaginal delivery [3].

An analysis by Boerma and colleagues [1] highlights the growing national inequities between LMICs with excessively high c-section rates and those whose low rates indicate insufficient access to care. However, the analysis only considers national c-section rates against the WHO threshold of 10%-15%. The study does not address potential sub-national variation in c-section use. In populations where c-section access may be variable, the use of elective c-sections by more advantaged subpopulations may inflate national c-section rate estimates and mask low coverage in the broader population. In the absence of data on the medical necessity of c-section births, it is difficult to disentangle the dual risks of c-section under and overuse.

To address the limitations presented by analyzing national c-section rates, we developed an approach to generate c-section coverage estimates to account for the potential inflating effect of c-section overuse (ie, elective c-sections) in subnational populations. Given the well-documented associations between household wealth and health intervention coverage [4,5], we stratified c-section rates by wealth quintile to adjust for the contribution of elective c-section in wealthier subnational populations. We assume that non-medically required c-sections are rare in low-income groups. Our analysis presents the relationship between wealth and c-section use, gauges the performance of our approach for estimating c-section coverage, and demonstrates the issues related to using c-section rate alone to characterize c-section utilization.

## METHODS

### Data sources and indicators

Our analysis used nationally representative household surveys conducted in low- and middle-income countries, including the Demographic and Health Survey (DHS: https://dhsprogram.com/) and Multiple Indicator Cluster Survey (MICS: mics.unicef.org). We restricted our analysis to the most recent survey conducted in each country and excluded countries whose most recent survey occurred before 2010, resulting in the inclusion of 98 countries.

Our analysis addresses disparities in c-section coverage by wealth and generates national coverage estimates accounting for variations in coverage and birth rates by wealth quintile. Both the DHS and MICS stratify their data using country-specific estimates of household wealth. Household wealth is estimated using a composite measure of each household's cumulative living standard based on ownership of select assets, home construction, and access to water and sanitation facilities. Each survey's wealth index is derived using a principal component analysis (PCA) to weight items by their relative importance in ascertaining an underlying construct of wealth. Surveyed households are assigned to one of five wealth quintiles (1 = poorest, 5 = wealthiest) of equal sample size based on their country and survey-specific wealth index score [6].

The DHS and MICS estimate c-section rates based on women's report of their most recent live birth in the past two years (MICS) or three years (DHS). Prior to round 8, the DHS collected data on live births in the past five years. We have restricted the analysis to the most recent live birth in the past three years. Additionally, the most recent DHS surveys capture data on both live births and stillbirths. However, this analysis does not include data from the most recent survey round and represents c-section rates among live births. Interviewers ask mothers if the baby was “delivered by cesarean, that is, did they cut your belly open to take the baby out?” The c-section rate among births in the last two or three years is estimated at the national and representative subnational levels, accounting for the survey sample design, including c-section rate within each wealth quintile. In determining c-section coverage, it is important to ascertain a denominator of c-section need. However, there are no questions in either the DHS or MICS for assessing whether a c-section was medically necessary or elective.

The crude birth rate was estimated for each survey by wealth quintile. The crude birth rate (CBR) represents the average number of births per thousand population within the specific wealth quintile. The CBR is calculated by summing the product of the current age-specific fertility rates and the proportion of women in the specific age group out of the total survey population [7].

We extracted national maternal mortality ratio (MMR), stillbirth rates (SBR), and World Bank income classification in the relevant survey year from external data sources. MMR data were sourced from UNICEF [8], SBR data from IGME [9], and historical data on World Bank classification were accessed through the World Bank [10]. MMR was used to define each country’s stage in the obstetric transition by survey year binned as >1000 maternal deaths per 100 000 live birth (stage 1), 999-300 deaths per 100 000 births (stage 2), 299-50 deaths per 100 000 births (stage 3), and <50 maternal deaths per 100 000 live birth (stage 4) [11].

### Calculating C-section coverage

In the absence of individual data to ascertain c-section need, we compared the national c-section rate against a threshold of appropriate c-section rate stratifying by wealth quintile. Assuming the 10%-15% of births necessitate c-sections, rates exceeding that threshold represent an excess of non-medically necessary c-sections. We used the midpoint of this range (12.5%) as our threshold for defining the proportion of births in need of c-section delivery. We assumed 12.5% of births within each wealth quintile should occur via c-section and used this estimate as our reference value for defining c-section need. If the stratum rate exceeded 12.5%, we capped the c-section coverage for that stratum at 100%. Rates less than 12.5% were converted to a proportion by dividing the rate by 12.5%.

**Figure 1:**
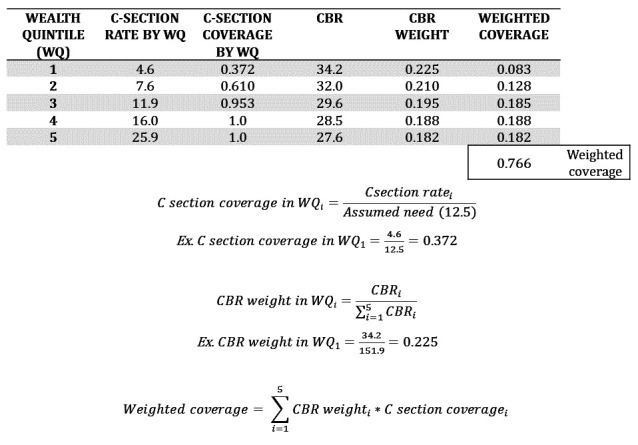
Example of the calculation for c-section formula.

We then calculated national c-section coverage by averaging the coverage across quintiles weighting for the quintile-specific relative CBR. Where CBR was unavailable by wealth quintile, each quintile was given equal weight. This process ensured the overall c-section coverage estimate reflected coverage among births in the total population. The example calculation is presented in **Figure 1**.

### Analysis

C-section coverage calculation and analyses were conducted in Stata 15 (StataCorp LLC, College Station TX, USA). We examined variation in estimated c-section coverage and c-section rate by wealth quintile, national income classification, and stage in the obstetric transition. We examined variations in c-section coverage and c-section rate accounting for the interaction between household wealth and national income classification by regressing c-section rate and c-section coverage on wealth quintile stratified by country income classification.

We created Equiplots [12] showing the c-section coverage within each quintile, the spread in coverage between quintiles, and how this relationship changes by underlying national c-section rate. We also graphically examined the relationship between c-section rate and c-section coverage at the national level and how varying thresholds and definitions around c-section rate and coverage impact the classification of countries’ access to c-section.

We assessed the face-validity of the c-section coverage estimates based on correlation with outcomes that are related to c-section. Lack of access to c-section can result in both maternal deaths and stillbirths, although other health interventions also affect these outcomes. We examined the strength of the association between c-section rate and c-section coverage and both MMR and SBR. We fit bivariate regression models to the data, exploring both linear and exponential growth relationships at a national level. We evaluated the fit of each model based on root mean square error (RMSE; a measure of the residual error between observations and the model), Akaike information criterion (AIC; an estimate of prediction error that weight model parsimony), and for linear models the coefficient of variation (R^2^; the proportion of variability explained by the independent variable).

We also calculated c-section coverage using c-section need thresholds of 10% and 15%. All analyses were reproduced using these alternative coverage measures and presented in the supplemental file. The results presented in the main text use the need threshold of 12.5%, but instances where use of the alternative threshold values substantially alter the results are noted.

## RESULTS

**Figure 2:**
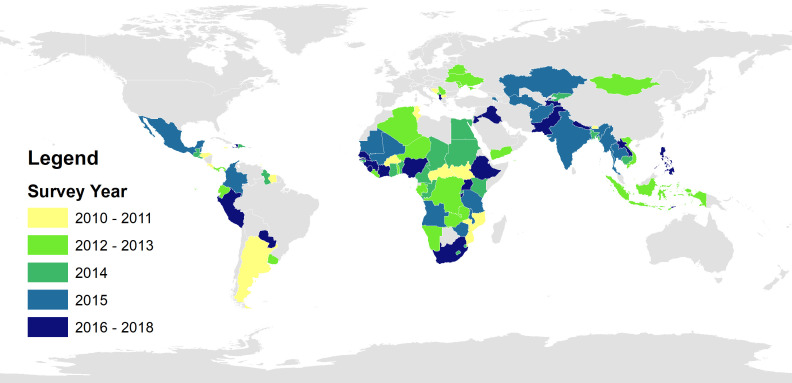
Survey data included in c-section coverage analysis.

**Figure 3:**
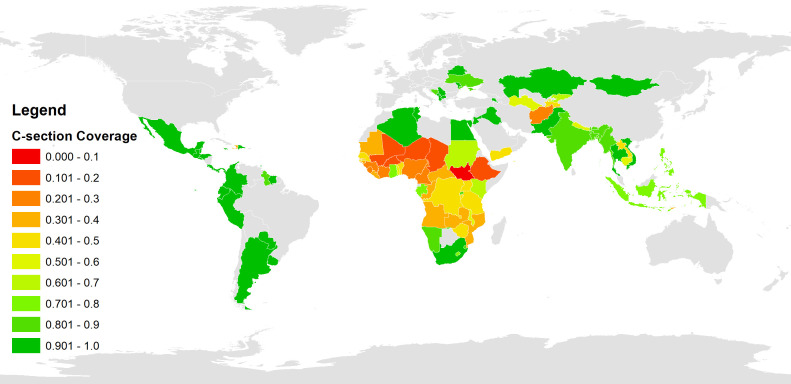
National c-section coverage.

DHS or MICS data on c-section rates by wealth quintile from 2010 or later were available for 98 countries. Most surveys were conducted in 2014 or later (**Figure 2**). This primarily included data on 68 countries defined by the World Bank as “middle-income” when the survey was conducted (**Table 1**). Based on MMR, more than two-thirds of the countries were at either stage 2 or stage 3 of the obstetric transition at the time of the survey. Countries with the lowest c-section coverage were clustered in sub-Saharan Africa, Central Asia, and Southeast Asia (**Figure 3**).

**Table 1:** National c-section rates and c-section coverage by within-country wealth quintile, World Bank income classification, and obstetric transition stage

	No.	C-section rate median (IQR)	C-section coverage median (IQR]
**C-section by wealth quintile**	98		
Q1		0.060 (0.021-0.153)	47.8% (16.9-100%]
Q2		0.094 (0.030-0.191)	75.0% (24.2-100%]
Q3		0.117 (0.038-0.235)	93.2% (30.5-100%]
Q4		0.143 (0.061-0.292)	100% (48.9-100%]
Q5		0.210 (0.121-0.378)	100% (96.9-100%]
Total population		0.128 (0.053 – 0.249)	81.4% (41.8-98.5%]
**C-section by World Bank income classification:**			
Low income	29	0.052 (0.031-0.065)	39.7% (24.7-47.4%]
Lower middle income	35	0.128 (0.056-0.204)	81.2% (42.2-96.6%]
Upper middle / high income	34	0.265 (0.190–0.334)	99.0% (96.6-100%]
**C-section by obstetric transition stage:**			
1 (MMR>1000)	4	0.024 (0.010-0.039)	19.6% (8.3-32.3%]
2 (MMR 999–300)	31	0.052 (0.031-0.067)	39.7% (24.7-50.6%]
3 (MMR 299–50)	37	0.185 (0.105-0.249)	86.9% (74.0-98.2%]
4 (MMR<50)	26	0.265 (0.193-0.315)	99.2% (97.0-100%]

IQR – interquartile range, MMR – maternal mortality ratio

### Relationship between wealth and c-section

There is a clear association between increasing wealth and both increasing c-section rates and c-section coverage (**Table 1**; Table S1 in the **Online Supplementary Document**). Across the 98 countries included in the analysis, the median c-section rate increased from 6% in the country's poorest wealth quintile to 21% in the wealthiest quintile. Similarly, the median c-section coverage ranged from 47.8% to 100% in the poorest and richest quintiles, respectively. Nationally, low- and middle-income countries experienced lower c-section rates and coverage.

An ecological analysis of the interaction between national and household wealth and c-section demonstrates the variation in how individual wealth is associated with c-section in countries with higher or lower national wealth (**Table 2**; Table S2 in the **Online Supplementary Document**). In low-income settings, we observe no statistically significant variation in c-section rates or coverage within the three lowest wealth quintiles, however, c-sections are significantly higher in the wealthiest quintiles. In low-middle income settings, the spread in c-section rates and coverage are more pronounced with the poorest household experiencing significantly lower c-sections rates and coverage, and the wealthiest quintiles experiencing significantly greater c-sections compared to the middle quintile by country. In upper-middle income settings, the spread in c-sections rates between the poorest and wealthiest is even more pronounced. However, c-section coverage estimates are less varied as coverage is relatively high among even the poorest households and there is a ceiling effect as coverage cannot exceed 100%. Figure S1 in the **Online Supplementary Document** shows the Equiplots of c-section coverage by World Bank classification.

**Table 2:** Variation in c-section rate and c-section coverage by wealth quintile within national income classification groups

	C-section rate			C-section coverage		
**National and household wealth classification**	**Coefficient**	**Coefficient 95% CI**	**Predicted rate**	**Coefficient**	**Coefficient 95% CI**	**Predicted coverage**
**Low income**						
Q1	-0.01	(-0.03, 0.00)	0.023	-0.12	(-0.23, 0.00)	0.184
Q2	-0.01	(-0.03, 0.01)	0.030	-0.06	(-0.17, 0.06)	0.243
Q3	Ref	-	0.038	Ref	-	0.302
Q4	0.02	(0.00, 0.04)	0.058	0.15	(0.04, 0.27)	0.456
Q5	0.08	(0.06, 0.10)	0.121	0.48	(0.37, 0.60)	0.784
**Lower middle income**						
Q1	-0.07	(-0.12, -0.01)	0.082	-0.24	(-0.38, -0.10)	0.507
Q2	-0.04	(-0.09, 0.02)	0.109	-0.11	(-0.25, 0.04)	0.639
Q3	Ref	-	0.147	Ref	-	0.744
Q4	0.04	(-0.02, 0.10)	0.188	0.08	(-0.06, 0.22)	0.825
Q5	0.11	(0.06, 0.17)	0.261	0.19	(0.05, 0.34)	0.938
						
**Upper middle / high income**						
Q1	-0.09	(-0.16, -0.02)	0.194	-0.07	(-0.14, 0.01)	0.886
Q2	-0.05	(-0.12, 0.02)	0.234	-0.02	(-0.10, 0.06)	0.931
Q3	Ref	-	0.286	Ref	-	0.952
Q4	0.03	(-0.04, 0.10)	0.318	0.01	(-0.07, 0.09)	0.962
Q5	0.11	(0.04, 0.18)	0.392	0.03	(-0.04, 0.11)	0.986

Q – quintile, CI – confidence interval

### Relationship between c-section rates and coverage

**Figure 4:**
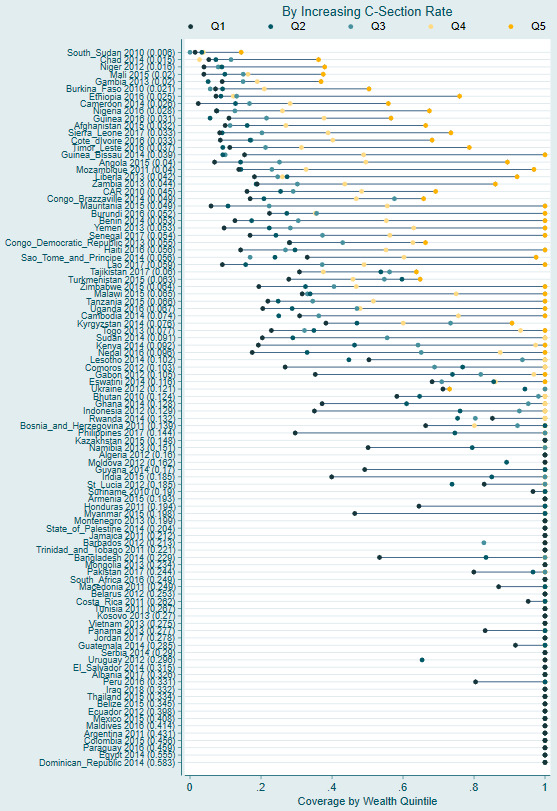
C-section coverage by wealth quintile, sorted on increasing national c-section rate.

**Figure 4** shows the spread in c-section coverage by wealth quintile sorted by c-section rate. At low c-section rates, such as observed in South Sudan, the spread in c-section coverage by household wealth is limited. Even the highest wealth quintiles lack access to c-section with only 15% coverage in the highest wealth category and below 5% coverage in the rest of the population.

As the c-section rate increases, the inequity in c-section coverage grows. A high spread in coverage is visible in countries with a c-section coverage rate between 4% and 10%. Among these countries, coverage in the poorest quintile ranges from 10 to 50%, while coverage in the wealthiest quintile typically exceeds 90%. The average coverage by quintile in this c-section rate group ranges from 20.1% in the poorest quintile to 28.5%, 39.3%, 57.7%, and 92% in each increasing wealth quintile. This demonstrates exponentially increasing coverage with increasing household wealth. There are some exceptions, notably Central African Republic, Democratic Republic of the Congo, Tajikstan, and Turkmenistan, where the spread in coverage is less pronounced as coverage in the wealthier quintiles is lower.

A reduced spread in coverage is evident among countries with a c-section rate between 10%-15%. In these settings, most countries have coverage of less than 60% in the poorest wealth quintile; however, coverage in the middle quintile is approximately 90%. Among countries with c-section rates above 15%, there is little difference in coverage by wealth quintile with the average coverage in the poorest quintile above 90%. However, there are some countries with high overall c-section rates with notable differentials in coverage. In Bangladesh, India, Guyana, Myanmar, and Namibia, coverage in the poorest quintile was below 60% despite national c-section rates above 15%.

Compared to a 12.5% need threshold, using a threshold of 10% we see a greater disparity in coverage by wealth within countries with c-section rates below 4% (Figure S2 in the **Online Supplementary Document**). Conversely, using a threshold of 15%, we see significant inequities in coverage among some nations with c-section rates in the 10%-15% range (Figure S3 in the **Online Supplementary Document**).

### Classification of access based on coverage vs rate

**Figure 5:**
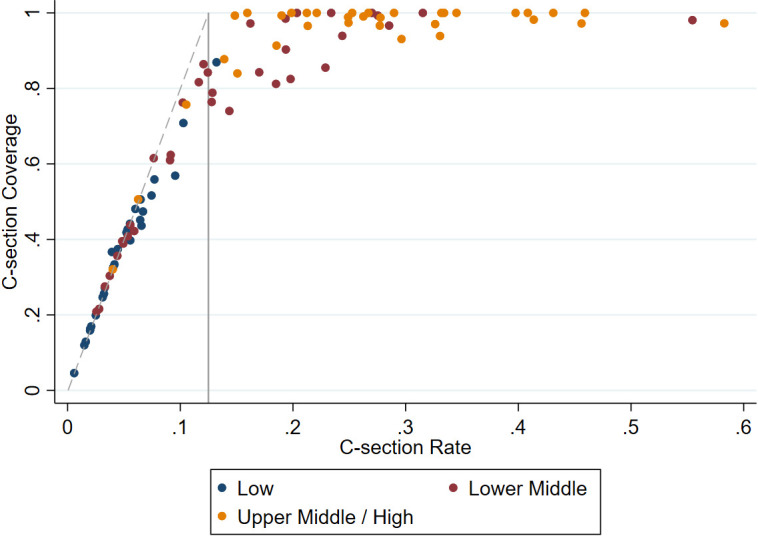
Relationship between c-section rate and c-section coverage at national level. Solid grey line indicates c-section rate threshold of 0.125. Dashed grey line represents a perfect relationship between c-section coverage and c-section rate, assuming 12.5% of births necessitate c-section.

**Figure 5** shows the deviation between c-section rates and c-section coverage. Numerous countries exceed a c-section rate threshold of 12.5% but have c-section coverage that falls below 100%. If all c-sections were medically indicated and equitably accessible, then a c-section rate of between 10%-15% would indicate sufficient coverage within the population. Using the cut-off of a 10% c-section rate, 56 of the 98 countries included in this analysis would meet this threshold. Using the upper limit of the range, 50 out of 98 countries have a c-section rate exceeding 15%.

However, when calculating coverage using a threshold of 12.5%, only 18 out of the 98 countries had a 100% c-section coverage. Forty countries had coverage above 90%, and 35 countries had coverage above 95%. As expected, if the threshold of need is reduced to 10% when calculating coverage, a greater proportion of countries achieve 90% (47) and 100% (23) coverage. With a higher 15% threshold of need, a lower proportion of countries achieve 90% (37) and 100% (15) coverage.

### Relationship between coverage and mortality

**Figure 6:**
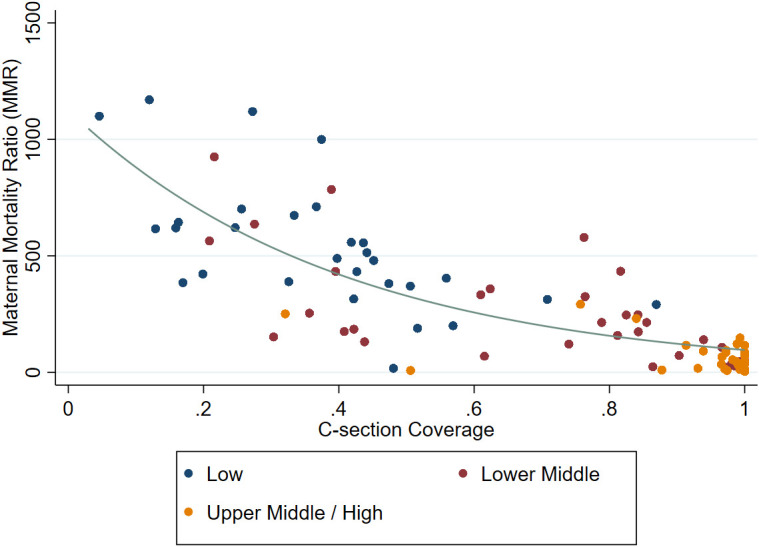
Relationship between national c-section coverage and maternal mortality ratio. Blue line indicates the best-fitting relationship (negative exponential regression) between MMR and c-section coverage.

**Figure 7:**
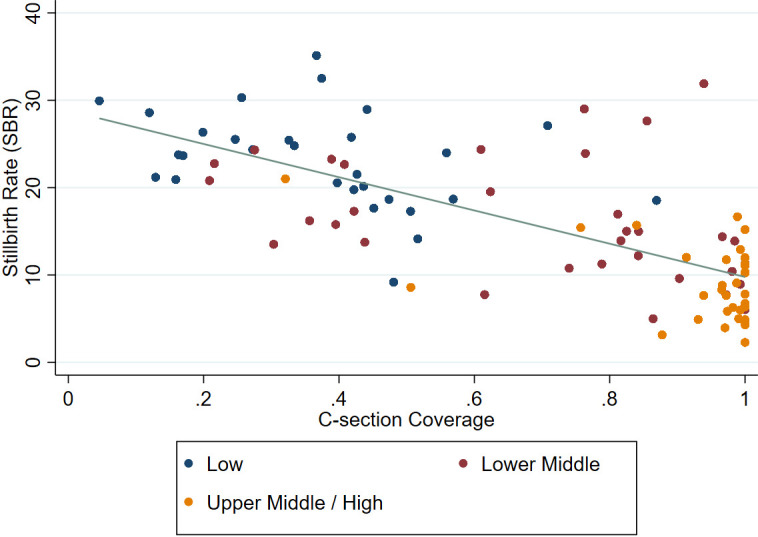
Relationship between national c-section coverage and stillbirth rate. Blue line indicates the best-fitting relationship (negative linear regression) between SBR and c-section coverage.

Both national estimates of stillbirths and maternal mortality show a clear relationship with c-section coverage. **Figure 6** shows the relationship between national c-section coverage and MMR. There is clear negative exponential relationship between increasing c-section coverage and decreasing MMR at a national level. Assuming a linear relationship between c-section and MMR, c-section coverage accounts for over 60% of the variation in national MMR and is a stronger predictor of MMR than c-section rate (Table S3 in the **Online Supplementary Document**). Assuming an exponential relationship, c-section coverage remains more strongly associated with MMR than c-section rate. **Figure 7** shows a linear relationship between increasing c-section coverage and decreasing SBR at a national level. C-section coverage explains approximately half of the variation in national SBR, while c-section rate only accounts for 38% of the variance (Table S3 in the **Online Supplementary Document**). Similar fit performance was observed using c-section coverage calculated using alternative thresholds of need (Table S4 in the **Online Supplementary Document**).

## DISCUSSION

This analysis shows a clear relationship between c-section coverage and wealth at both a household and ecological level. Both increasing national wealth and household wealth are associated with increasing levels of c-section coverage and c-section rate. The association between household wealth and c-section rates supports our approach of calculating stratified c-section coverage by quintile and capping coverage to adjust for elective c-sections in the wealthiest households. Differentials in coverage are most pronounced in countries with c-section rates below 10%; however, there are some countries where significant gaps in c-section coverage are present in poor subpopulations despite high c-section rates nationally. Looking at national c-section rates alone may paint an inaccurate picture of c-section access and mask subnational inequities in coverage. Our c-section coverage estimates showed a stronger relationship with closely related health outcomes (MMR and SBR) than national c-section rates.

National c-section rates mask inequities in access due to the contribution of elective c-sections in wealthier populations and overestimate coverage and access to c-section services. In the absence of data on whether an individual c-section was necessary, we are limited to comparing national c-section rates against a threshold of 10%-15% of births necessitating a c-section. However, excess elective c-sections in more advantaged populations can offset deficits in c-section access in disadvantaged populations when examined at an aggregate level.

The association between wealth and greater access to health interventions has been well documented [4,5]. Given the clear association between wealth and c-section rate presented here, generating estimates of c-section coverage stratifying by wealth quintile and capping coverage at 100% seems a reasonable approach to restrict the inflating effect of non-medically necessary c-sections in those populations where high rates indicate over-use. This approach produces coverage estimates that show a stronger association than c-section rate with the two health outcomes most affected by c-section. The strong ecological association between c-section coverage and both MMR and SBR supports the validity of our approach's coverage estimates.

In gauging countries with sufficient use of c-section services, coverage estimates show a bleaker picture of access compared to examining c-section rates alone. While over half of the countries included in this analysis had c-section rates exceeding 10%, only around a third had coverage levels above 95%. Subnationally, there are substantial inequities in coverage. Particularly in countries with national rates between 4%-10%, we see that coverage in the poorest quintiles averages below 30%. Even in countries with c-section rates in the 10%-15% range, only 60% of women in need in the poorest wealth quintiles access c-sections. Notably, some countries such as Bangladesh with c-section rates that would flag an excessive use of c-sections have coverage under 60% within their most impoverished populations.

This analysis supports our approach of estimating c-section coverage stratifying by wealth to account for the contribution of elective c-sections to the overall c-section rate. However, we lack data on whether individual c-sections were medically necessary. This ecological analysis could not adjust for potential elective c-sections that occurred in strata with rates below 12.5%. In these strata, some proportion of c-sections may be elective, and these events will inflate the observed coverage in the stratum despite lacking a denominator of need. As a result, our coverage estimates are most likely to overestimate c-section coverage in these populations.

Additionally, we applied a c-section rate threshold of 12.5% as our reference rate for defining need in the primary analysis. We selected 12.5% as the midpoint of the WHO guidance that 10%-15% of births should occur via a medically necessary c-section. However, this value does not account for potential variability in c-section need by population, as rates of high-risk pregnancies may be affected by other factors related to antenatal care, fertility trends, and medical risk factors [3,13]. To account for the potential variation in underlying need, we performed each analysis using alternative reference rates of 10% and 15% c-section need as sensitivity analyses and found no significant differences in results using these alternative cut-offs.

## CONCLUSIONS

Cesarean section is a crucial medical intervention for averting maternal deaths, intrapartum stillbirths, and early neonatal deaths. Although many countries have c-section rates that approach the threshold of 10%-15% c-section births, there is notable inequity in c-section coverage. National c-section rates mask subnational inequities in coverage in the poorest segments of the population. Further, elective c-sections in wealthier populations overinflate c-section rates and distort estimates of the proportion of the population that can access a medically necessary c-section. Generating estimates of c-section coverage that can more precisely assess c-section use among those in need is crucial for gauging gaps in c-section access. This is particularly relevant as continued growth in LMICs drives inequities in coverage and introduces dual concerns related to c-section overuse in some populations while others lack access to care. Our approach of using stratified analysis to adjust for elective c-section in wealthy quintiles appears to be a valid method for generating more robust estimates of c-section coverage. However, better data to understand populations at risk and disaggregate medically necessary and elective c-sections are needed as facility delivery rates continue to increase and the quality of care becomes increasingly relevant.

## Additional material


Online Supplementary Document
