# Supplementary material for: Estimating c-section coverage: Assessing method performance and characterizing variations in coverage
Source: J Glob Health. 2022 Apr 9;12:08002. doi: 10.7189/jogh.21.08002 (PMC8982632; doi:10.7189/jogh.21.08002)
Supplement: Online Supplementary Document [file jogh-12-08002-s001.pdf]

Supplementary material for:

Estimating c-section coverage: Assessing method performance and characterizing variations in coverage

Figure S1. C-section coverage by wealth quintile and World Bank income classification

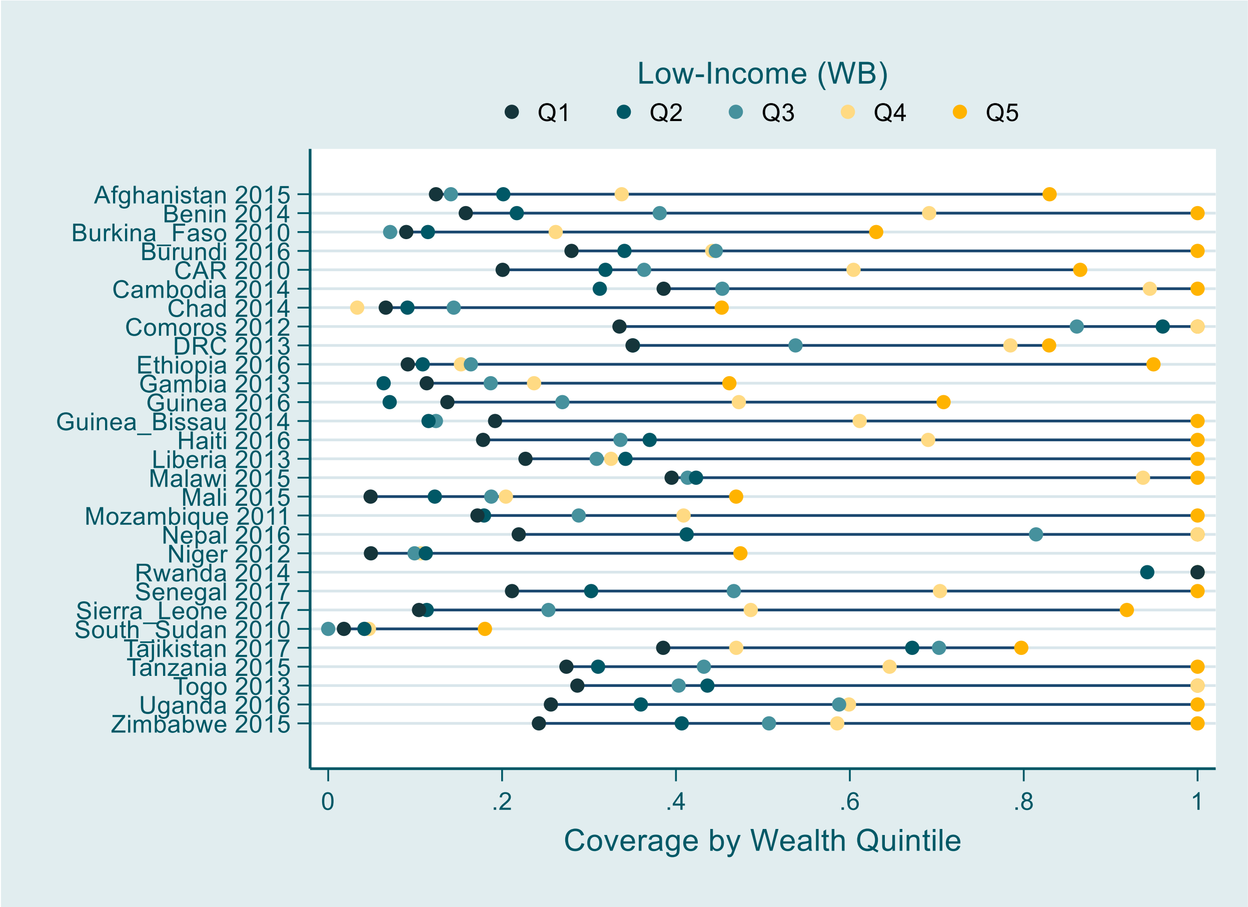

Figure S1. C-section coverage by wealth quintile and World Bank income classification, continued

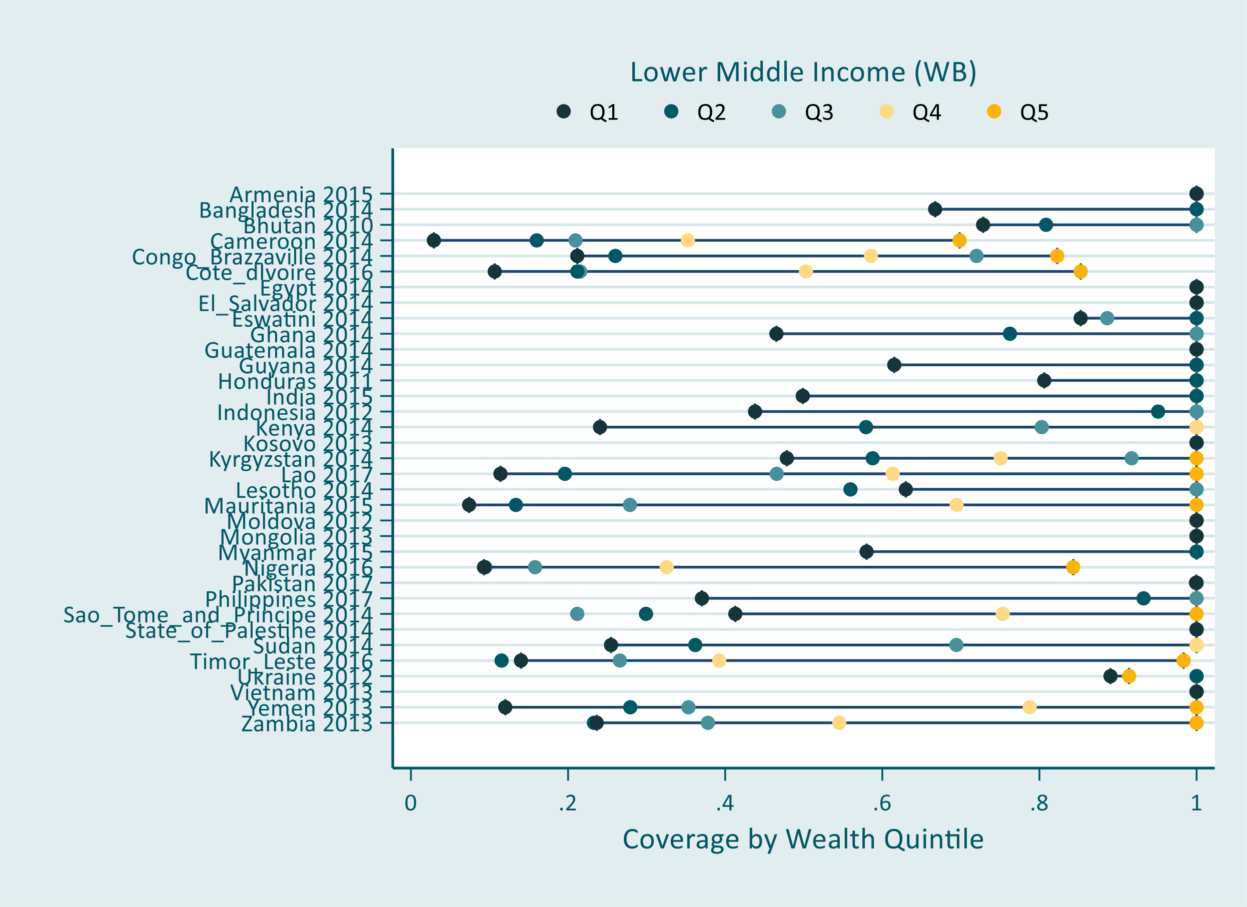

Figure S1. C-section coverage by wealth quintile and World Bank income classification, continued

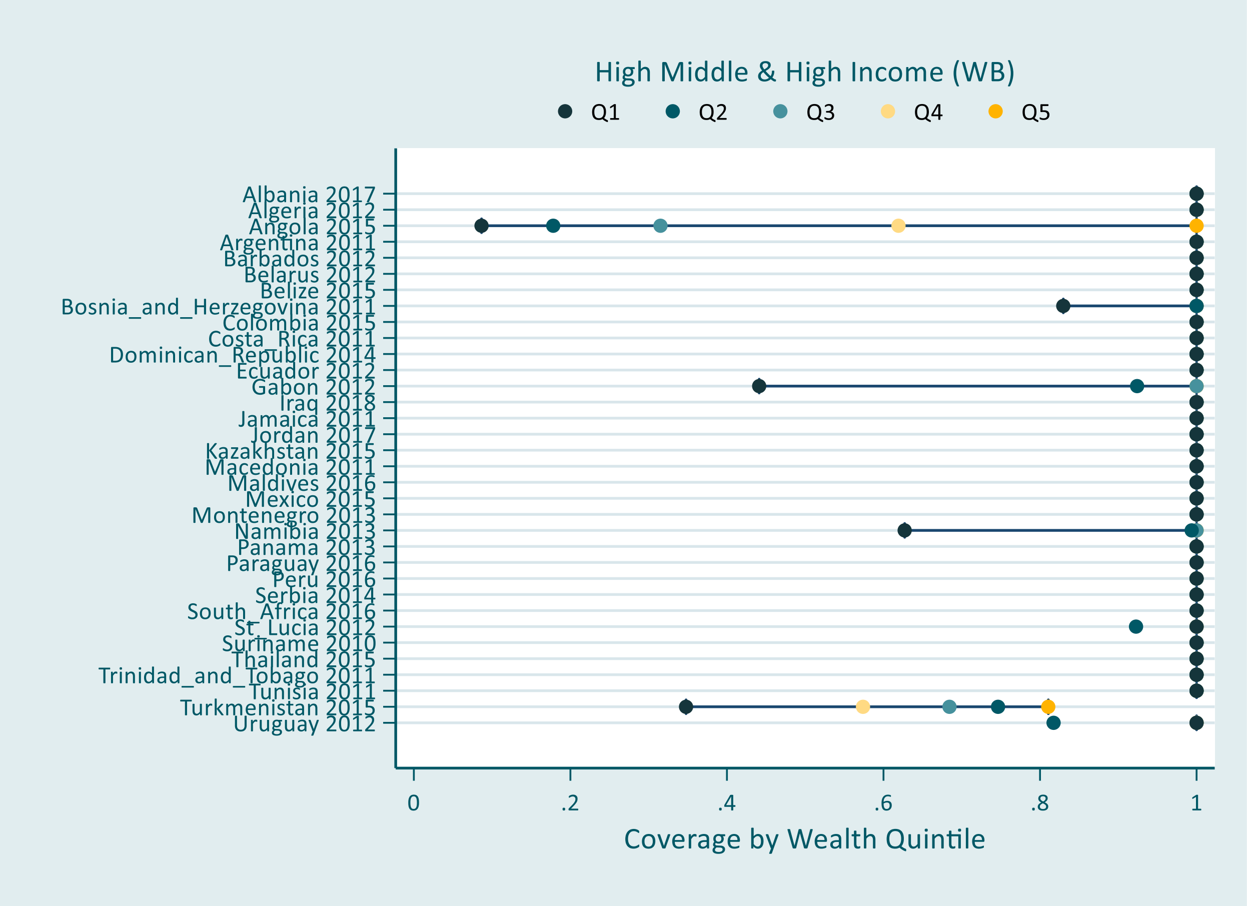

Table S1: National c-section coverage by within-country wealth quintile, World Bank income classification, and obstetric transition stage using alternative estimates of need

	n	**C-section Coverage 10% need** Median [IQR]	**C-section Coverage 15% need** Median [IQR]
**C-section by wealth quintile**	98		
Q1		59.8% [21.2 - 100%]	39.8% [14.1 - 100%]
Q2		93.7% [30.2 - 100%]	62.5% [20.2 - 100%]
Q3		100% [38.1 - 100%]	77.7% [25.4 - 100%]
Q4		100% [61.1 - 100%]	95.1% [40.8 - 100%]
Q5		100% [100 - 100%]	100% [80.7 - 100%]
Total Population			
**C-section by World Bank income classification**			
Low income	29	46.8% [30.8 - 55.2%]	35.0% [20.6 - 40.4%]
Lower middle income	35	86.0% [49.4 - 98.5%]	72.1% [36.5 - 92.5%]
Upper middle / high income	34	100% [97.2 - 100%]	97.1% [92.8 - 100%]
**C-section by obstetric transition stage**			
1 (MMR >1000)	4	24.5% [10.4 - 40.4%]	16.3% [6.9 - 27.0%]
2 (MMR 999–300)	31	48.0% [30.8 - 59.3%]	35.0% [20.6 - 43.0%]
3 (MMR 299–50)	37	94.3% [80.2 - 99.2%]	81.6% [65.7 - 97.2%]
4 (MMR <50)	26	100% [98.1 - 100%]	98.3% [93.4 - 100%]

Table S2. Variation in c-section coverage by wealth quintile within national income classification groups using alternative estimates of need

	C-section Coverage - 10% need			C-section Coverage – 15% need		
**Low income**	**Coefficient**	**Coefficient 95% CI**	**Predicted Coverage**	**Coefficient**	**Coefficient 95% CI**	**Predicted Coverage**
Q1	-0.15	(-0.28, -0.02)	22.7%	-0.10	(-0.20, 0.01)	15.3%
Q2	-0.07	(-0.20, 0.05)	30.4%	-0.05	(-0.15, 0.06)	20.2%
Q3	Ref	-	37.7%	Ref	-	25.2%
Q4	0.17	(0.04, 0.29)	54.4%	0.13	(0.03, 0.24)	38.6%
Q5	0.47	(0.34, 0.60)	84.7%	0.46	(0.35, 0.56)	71.0%
**Lower middle income**						
Q1	-0.21	(-0.35, -0.08)	57.3%	-0.23	(-0.38, -0.09)	46.1%
Q2	-0.09	(-0.33, 0.05)	70.1%	-0.11	(-0.26, 0.03)	58.1%
Q3	Ref	-	78.7%	Ref	-	69.5%
Q4	0.08	(-0.06, 0.21)	86.6%	0.09	(-0.06, 0.23)	78.1%
Q5	0.19	(0.05, 0.32)	97.5%	0.21	(0.06, 0.35)	90.2%
**Upper middle / high income**						
Q1	-0.05	(-0.12, 0.02)	92.2%	-0.09	(-0.18, -0.01)	83.7%
Q2	-0.01	(-0.08, 0.05)	95.8%	-0.02	(-0.11, 0.07)	91.0%
Q3	Ref	-	97.0%	Ref	-	93.0%
Q4	0.01	(-0.06, 0.07)	97.6%	0.02	(-0.07, 0.11)	94.9%
Q5	0.02	(-0.04, 0.09)	99.4%	0.05	(-0.04, 0.14)	97.8%

Table S3. Relationship between c-section rate and c-section coverage and health outcome (MMR and SBR) and goodness-of-fit measures.

	**C-section Rate**	**C-section Coverage**
**Maternal Mortality Ratio (MMR)**		
Exponential Relationship	$MMR=915.46 * 1.90e\text{-}06^{rate}$	**$MMR=1126.46 * 0.085^{coverage}$**
RMSE	169.3319	167.1956
AIC	997.560	995.097
Linear Relationship	MMR=492.48 – 1354.4 * rate	MMR=775.6 – 732.2 * coverage
R^2	0.4085	0.6324
Adjusted R^2	0.4022	0.6285
RMSE	216.700	170.830
AIC	1045.409	999.272
**Stillbirth Rate (SBR)**		
Exponential Relationship	$SBR=25.08 * 0.0319^{rate}$	$SBR=31.43 * 0.3402^{coverage}$
RMSE	5.9409	5.7741
AIC	347.660	342.136
Linear Relationship	SBR=21.90-37.69*rate	**SBR=28.73 – 18.85*coverage**
R^2	0.3849	0.5098
Adjusted R^2	0.3784	0.5046
RMSE	6.3347	5.6553
AIC	360.110	338.102

***best-fitting model noted in bold**

Table S4. Relationship between c-section coverage and health outcome (MMR and SBR) and goodness-of-fit measures using alternative estimates of need

	**C-section Coverage – 10% need**	**C-section Coverage – 15% need**
**Maternal Mortality Ratio (MMR)**		
Exponential Relationship	$MMR=1236.96 * 0.093^{coverage}$	$MMR=1051.44 * 0.075^{coverage}$
RMSE	169.032	166.609
AIC	997.216	994.415
Linear Relationship	MMR=844.73 – 781.48*coverage	MMR=723.95 – 698.36*coverage
R^2	0.641	0.620
Adjusted R^2	0.638	0.616
RMSE	168.740	173.700
AIC	996.886	1002.506
**Stillbirth Rate (SBR)**		
Exponential Relationship	$SBR=33.46 * 0.336^{coverage}$	$SBR=30.08 * 0.338^{coverage}$
RMSE	5.847	5.701
AIC	344.573	339.653
Linear Relationship	SBR=30.28 – 19.80*coverage	SBR=27.60 – 18.29*coverage
R^2	0.501	0.517
Adjusted R^2	0.496	0.512
RMSE	5.705	5.612
AIC	339.795	336.597

Figure S2. C-section coverage by wealth quintile, sorted on increasing national c-section rate, coverage calculated based on 10% c-section need

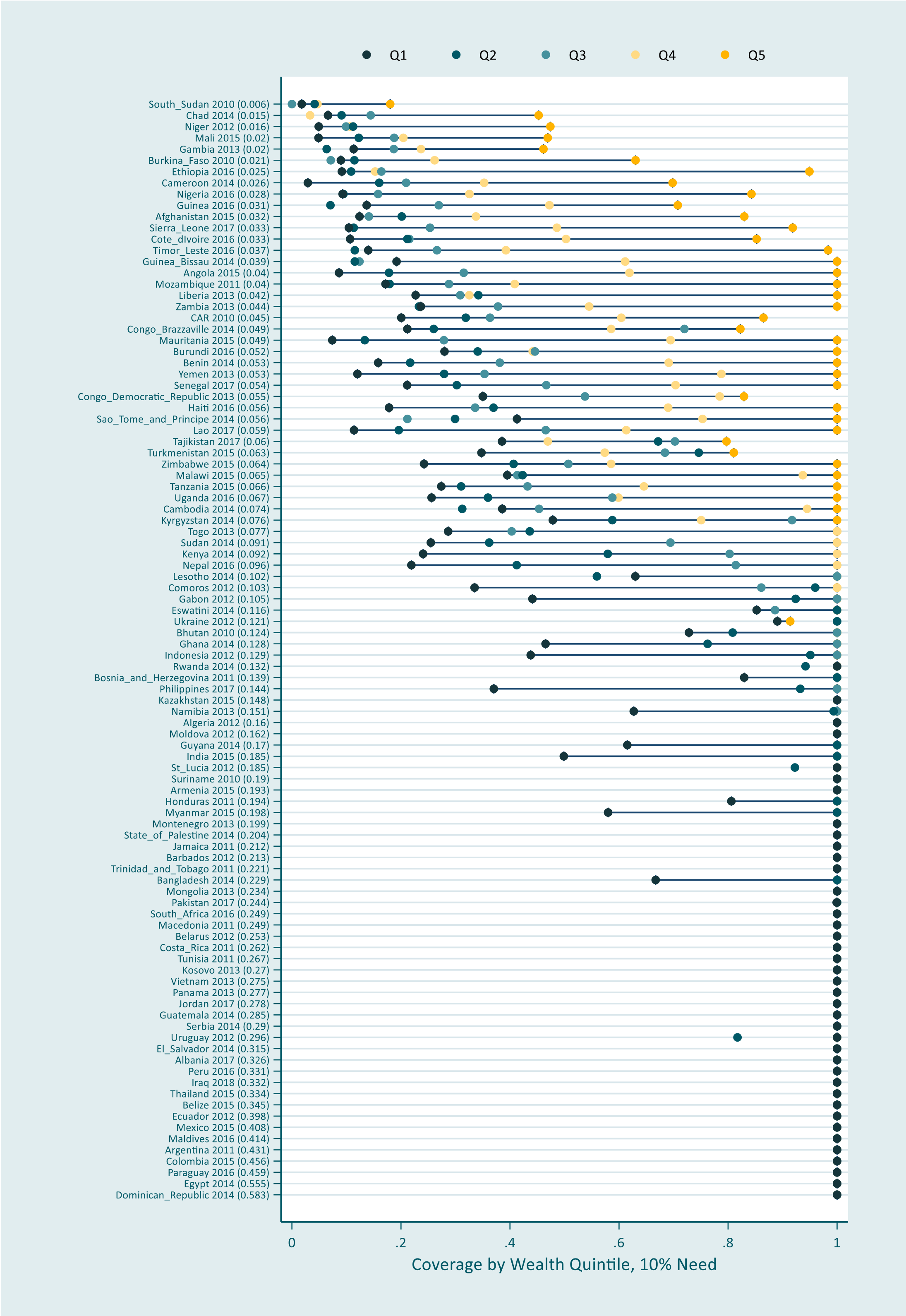

Figure S3. C-section coverage by wealth quintile, sorted on increasing national c-section rate, coverage calculated based on 15% c-section need

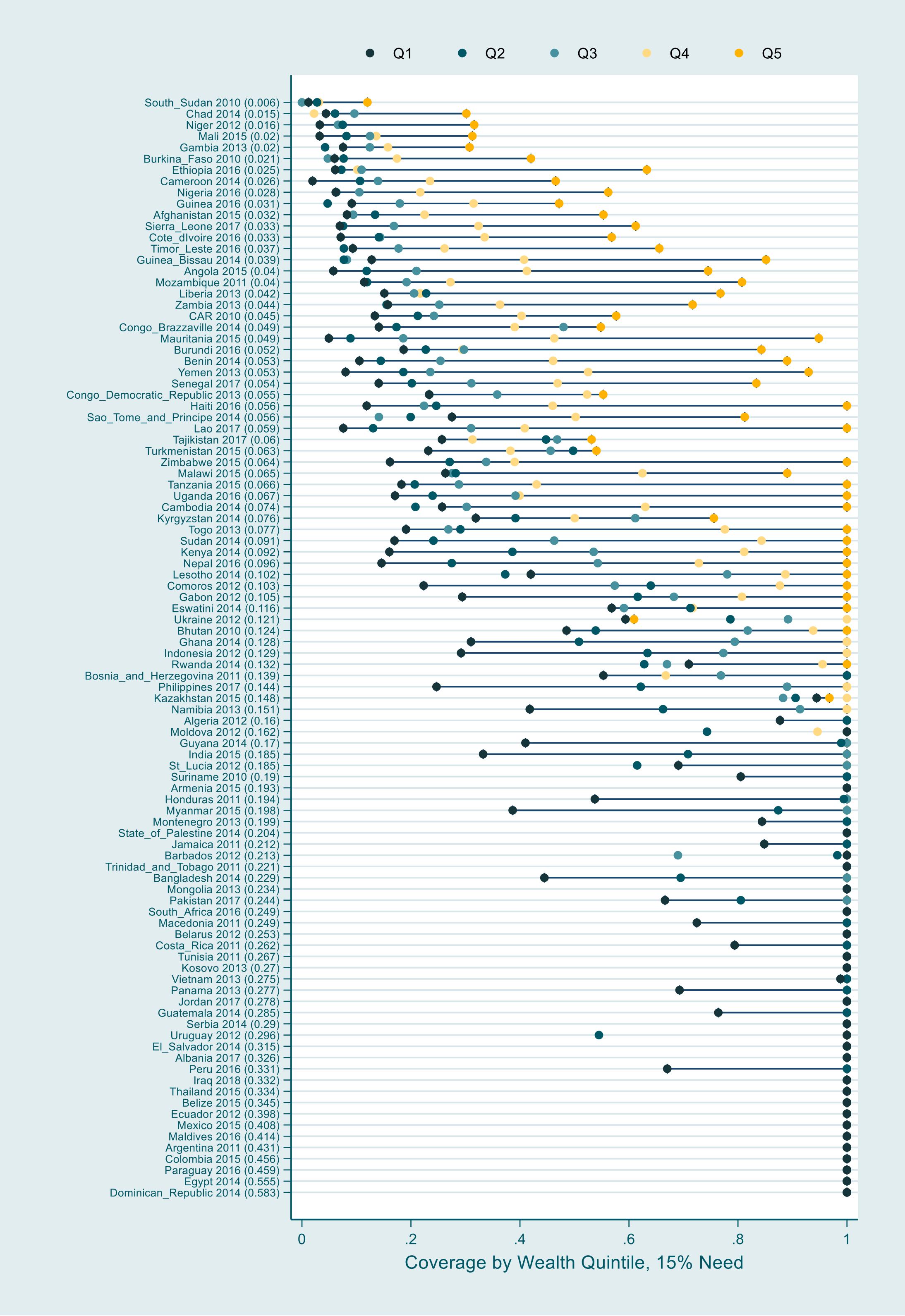

Figure S4. Relationship between c-section rate and c-section coverage at national level using alternative estimates of need

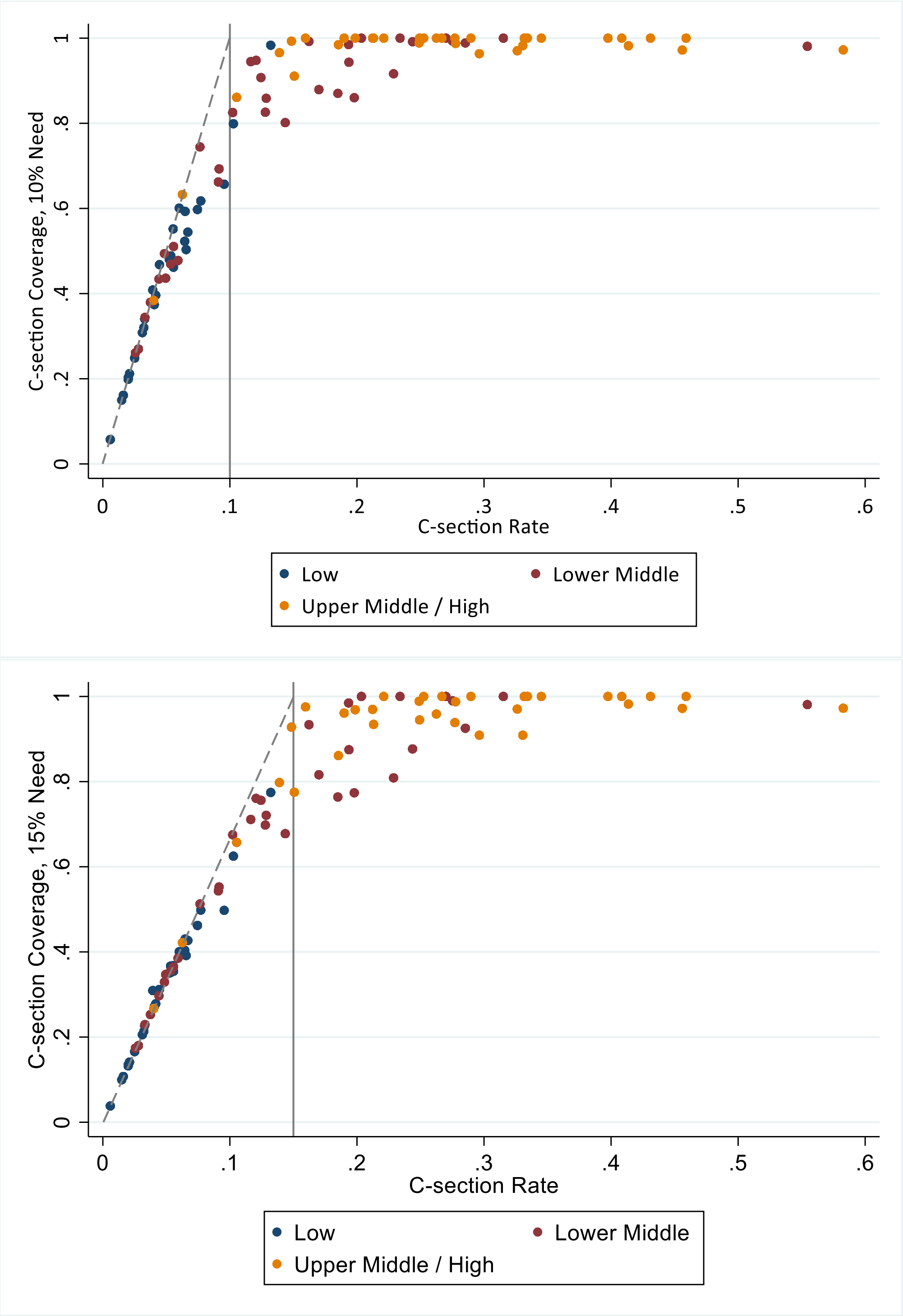

Solid grey line indicates c-section rate threshold. Dashed grey line represents a perfect relationship between c-section coverage and c-section rate.
